# Multimodal biomarkers of response to transcutaneous auricular vagus nerve stimulation in disorders of consciousness: a study protocol

**DOI:** 10.3389/fnhum.2026.1868162

**Published:** 2026-09-16

**Authors:** Weihang Zhai, Yifei Wang, Meng Li, Yi Yang, Jinling Zhang, Jianghong He, Peijing Rong

**Affiliations:** 1 Institute of Basic Research in Clinical Medicine, China Academy of Chinese Medical Sciences, Beijing, China; 2 Institute of Acupuncture and Moxibustion, China Academy of Chinese Medical Sciences, Beijing, China; 3 Department of Trauma Surgery, Zhengzhou Central Hospital Affiliated to Zhengzhou University, Zhengzhou, China; 4 Department of Neurosurgery, Beijing Tiantan Hospital, Capital Medical University, Beijing, China

**Keywords:** disorders of consciousness, electroencephalography, functional near-infrared spectroscopy, multimodal biomarkers, transcutaneous auricular vagus nerve stimulation

## Abstract

Patients with disorders of consciousness (DOC) have limited options for arousal-promoting interventions. Transcutaneous auricular vagus nerve stimulation (taVNS), a non-invasive neuromodulation technique, has shown potential to improve conscious behaviors in a subset of patients with DOC. However, substantial interindividual variability in treatment response remains, and reliable objective biomarkers for identifying patients who may benefit are still lacking. This study is designed as a single-center, prospective, exploratory clinical study. A total of 50 patients with DOC will be consecutively recruited from the Neurosurgical Ward for DOC at Beijing Tiantan Hospital, Capital Medical University. All enrolled patients will receive 4 weeks of taVNS treatment in addition to standard rehabilitation. Assessments using the Coma Recovery Scale-Revised (CRS-R), electroencephalography (EEG), and functional near-infrared spectroscopy (fNIRS) will be performed at baseline, week 2, and week 4 of treatment. The primary outcome will be the observed change in the CRS-R total score from baseline to week 4. Secondary outcomes will include changes in CRS-R subscale scores, diagnostic transitions in consciousness state, and prespecified EEG and fNIRS features associated with treatment response. Responders will be defined as patients with an increase of at least 3 points in the CRS-R total score from baseline to week 4. Because this is an uncontrolled single-arm study, the findings will be interpreted as exploratory and hypothesis-generating rather than as evidence of causal efficacy. This study aims to characterize behavioral, electrophysiological, and hemodynamic changes during taVNS plus standard rehabilitation and to identify candidate multimodal biomarkers associated with observed improvement, thereby providing a methodological basis for future randomized controlled trials in this population.

**Clinical trial registration:**http://itmctr.ccebtcm.org.cn/en-US/ITMCTR2024000734, identifier ITMCTR2024000734

## 1 Background

Disorders of consciousness (DOC) refer to a clinical condition in which impaired consciousness persists for more than 28 days after severe brain injury, mainly including unresponsive wakefulness syndrome/vegetative state (UWS/VS) and minimally conscious state (MCS) (Giacino et al., 2018a; Giacino et al., 2002; Laureys et al., 2010).

The treatment of DOC has long remained a major focus and challenge in clinical intervention (Giacino et al., 2014). At present, arousal-promoting therapies for patients with DOC mainly include surgical neuromodulation approaches such as spinal cord stimulation (SCS) and deep brain stimulation (DBS) (Thibaut et al., 2019). Previous studies have shown that these surgical interventions may improve arousal levels and behavioral responsiveness in some patients, offering a new therapeutic direction for the management of disorders of consciousness (Xia et al., 2018; Lemaire et al., 2014). However, invasive treatments such as SCS and DBS usually require strict preoperative evaluation and surgical support, and are associated with risks related to anesthesia, postoperative infection, and multiple complications. In addition, these therapies are costly and resource-intensive, making them unaffordable for many families and preventing a substantial proportion of patients with DOC from receiving effective treatment (Zheng et al., 2023; Young et al., 2021).

In contrast, transcutaneous auricular vagus nerve stimulation (taVNS), as a non-invasive neuromodulation technique, has attracted increasing attention in the field of DOC in recent years because of its advantages of relatively high safety, ease of operation, repeatability, and comparatively low treatment cost (Yap et al., 2020). By stimulating the auricular regions innervated by the vagus nerve, taVNS may modulate thalamocortical networks associated with consciousness, thereby promoting improvement in patients’level of consciousness (Butt et al., 2020; Frangos et al., 2015). Existing studies have shown that taVNS can improve Coma Recovery Scale-Revised (CRS-R) scores and facilitate diagnostic transition in some patients with DOC, suggesting its promising clinical potential (Zhou et al., 2023; Yu et al., 2017). Particularly for patients who are bedridden for long periods, have complex medical conditions, and place a heavy economic burden on their families, taVNS may represent a more accessible arousal-promoting intervention (Jang and Cho, 2022).

However, it should be noted that not all patients with DOC benefit from taVNS treatment. Considerable interindividual variability exists in clinical practice: some patients show a favorable therapeutic response, whereas others demonstrate little or no obvious improvement (Dong et al., 2023; Yu et al., 2021). Therefore, identifying the characteristics of patients with DOC who are more likely to benefit from taVNS, and screening potential responders, has become a key issue that urgently needs to be addressed (Zhai et al., 2023; Vitello et al., 2023).

Electroencephalography (EEG), with its high temporal resolution, bedside applicability, and relatively low cost, has become one of the most commonly used neurophysiological assessment tools in DOC research (Wutzl et al., 2021). Previous studies have shown that EEG can reflect brain functional status in patients with DOC at multiple levels, including spectral power, functional connectivity, and network complexity, and is of important value for the detection of residual consciousness, disease stratification, and prognostic evaluation (Szirmai et al., 2024; Naro et al., 2018; Sitt et al., 2014; Ballanti et al., 2022). In particular, for some patients in whom behavioral assessments may not accurately reflect their true level of consciousness, EEG can provide objective evidence for the detection of covert consciousness (Jain and Ramakrishnan, 2020; Forgacs et al., 2014).

Functional near-infrared spectroscopy (fNIRS) is a non-invasive, portable, and bedside-applicable neuroimaging technique that can indirectly reflect cortical neural activity and cerebral hemodynamic status by measuring changes in the concentrations of oxygenated and deoxygenated hemoglobin in brain tissue (Rupawala et al., 2018; Kempny et al., 2016). In recent years, the application of fNIRS in the field of DOC has gradually increased. Studies have shown that it can be used to assess activation levels, functional connectivity, and responses to external stimuli in the prefrontal and other cortical regions, and may provide valuable information for evaluating residual cognitive processing and level of consciousness (Li et al., 2021; Liu et al., 2023; Molteni et al., 2013; Kurz et al., 2018). Compared with EEG, fNIRS can complement brain functional assessment from a hemodynamic perspective, compensating for the limitations of single electrophysiological indicators in spatial localization and metabolic characterization, and thus providing a new objective approach for evaluating brain function in patients with DOC (Lu et al., 2023; Si et al., 2023).

The *a priori* selection of EEG and fNIRS features was guided by the Global Neuronal Workspace framework and the frontoparietal network disruption hypothesis of impaired consciousness. The Global Neuronal Workspace framework proposes that conscious access depends on recurrent large-scale integration and global broadcasting of information across distributed cortical and thalamocortical systems (Mashour et al., 2020). Consistent with this account, long-distance information sharing measured by EEG increases with the level of consciousness in noncommunicative patients (King et al., 2013), and previous DOC studies have identified theta/alpha spectral organization, functional connectivity, and signal complexity as candidate indicators of preserved or recovering consciousness (Sitt et al., 2014). Accordingly, frontal and parietal theta/alpha power, frontoparietal weighted phase-lag connectivity, global efficiency, and Lempel-Ziv complexity were prespecified as the principal EEG candidate features.

For fNIRS, the prefrontal cortex was selected as an accessible cortical component of large-scale integrative networks, whereas the primary motor and sensorimotor cortices were included because motor-imagery and sensorimotor paradigms may reveal residual command-related cortical processing when overt behavioral output is limited (Li et al., 2021; Liu et al., 2023). Direct fNIRS evidence during taVNS in DOC remains limited; however, a recent case report assessed taVNS-associated changes in frontoparietal connectivity using fNIRS channels covering the prefrontal, primary motor, and primary sensory cortices (Gao et al., 2025), while previous taVNS neuroimaging studies demonstrated modulation of distributed cerebral hemodynamic and functional networks (Yu et al., 2017; Yu et al., 2021). Thus, these regions and features were selected *a priori* for hypothesis-driven feature screening rather than for exhaustive data-driven discovery.

Based on these considerations, the present study proposes to combine EEG and fNIRS for multimodal assessment of patients with DOC from the two dimensions of electrophysiological activity and cerebral hemodynamics (Wang et al., 2025). By integrating clinical behavioral changes before and after taVNS treatment, this study aims to identify objective brain functional features associated with observed treatment response and to explore candidate multimodal biomarkers that may help identify patients with DOC who are more likely to show improvement during taVNS plus standard rehabilitation. By constructing a comprehensive evaluation framework that integrates clinical scales with neurophysiological and hemodynamic indicators, we hope to provide preliminary evidence for individualized intervention strategies and for the design of future controlled studies (Gallucci et al., 2024; Kondziella et al., 2020; Giacino et al., 2018b; Edlow et al., 2021).

## 2 Study aims

This study aims to characterize behavioral changes during a 4-week course of taVNS plus standard rehabilitation in patients with DOC and to identify multimodal brain functional biomarkers associated with observed treatment response by integrating EEG and fNIRS. The study population was selected for three main reasons. First, arousal-promoting treatment options for DOC remain limited. Although neuromodulation approaches such as SCS and DBS have shown therapeutic benefit in a subset of patients, they are invasive interventions associated with substantial surgical risks, high costs, and limited clinical accessibility. Second, taVNS, as a non-invasive, convenient, and relatively low-cost neuromodulation approach, has demonstrated potential to improve conscious behavior in some patients with DOC; however, treatment effects vary considerably across individuals, highlighting the need to identify those who are more likely to show improvement. Third, EEG and fNIRS reflect brain functional status from the perspectives of electrophysiological activity and cerebral hemodynamics, respectively. Their complementary strengths may enable a more comprehensive characterization of residual consciousness and treatment-related brain functional changes.

This exploratory study is expected to provide a more precise evaluation framework for taVNS in patients with DOC by identifying differences in multimodal brain functional biomarkers between responders and non-responders. Specifically, this study will evaluate changes in CRS-R scores during taVNS plus standard rehabilitation and will further explore potential neural correlates and candidate multimodal biomarkers of treatment response by integrating CRS-R scores, EEG, and fNIRS measures. These findings are intended to support the design of future large-scale randomized controlled trials and to inform individualized arousal-promoting intervention strategies in patients with DOC.

## 3 Primary research objective

The primary objective of this study is to characterize the observed change in the CRS-R total score from baseline to week 4 during taVNS plus standard rehabilitation in patients with DOC and to identify multimodal brain functional biomarkers associated with taVNS response at the end of treatment.

## 4 Secondary research objective

The secondary objectives of this study are to further evaluate the following aspects in patients with DOC:

1) Changes in CRS-R total scores and subscale scores at baseline, week 2, and week 4;
2) Post-treatment diagnostic transitions in the state of consciousness, including transitions from UWS/VS to MCS-, or from UWS/VS or MCS- to MCS+;
3) Changes in prespecified EEG features between the responder and non-responder groups, including spectral power in conventional frequency bands, functional connectivity, network complexity, and microstate parameters. We hypothesize that responders will show relatively preserved or increased frontoparietal network activity and higher network integration over the treatment course;
4) Changes in prespecified fNIRS features between the responder and non-responder groups, including oxygenated and deoxygenated hemoglobin responses, regional activation patterns, and functional connectivity over the prefrontal, motor, and sensorimotor cortices. We hypothesize that responders will show stronger resting-state hemodynamic fluctuations and more preserved cortical functional connectivity.

## 5 Methods and design

### 5.1 Study design

This study is designed as a single-center, prospective, exploratory clinical study. Patients with DOC will receive a 4-week taVNS intervention in addition to standard rehabilitation, and will undergo CRS-R, EEG, and fNIRS assessments at baseline, week 2, and the end of treatment. Because no sham-control, randomization, or standard-care-only comparison group is included, the study is not designed to determine the causal efficacy of taVNS. Instead, it is intended to characterize behavioral changes during taVNS plus standard rehabilitation and to identify candidate multimodal brain functional biomarkers associated with observed response.

### 5.2 Ethics approval and clinical trial registration

This study has been approved by the Ethics Committee of the Institute of Acupuncture and Moxibustion, China Academy of Chinese Medical Sciences (2022-05-25), and has been registered with the International Traditional Medicine Clinical Trial Registry (ITMCTR2024000734). Because patients with DOC are unable to provide informed consent independently, written informed consent will be obtained from the legal representative of each participant before enrollment.

### 5.3 Inclusion and exclusion criteria

Inclusion criteria: (1) diagnosis of DOC; (2) age between 18 and 70 years; (3) disease duration ≥3 months; (4) negative pregnancy test in female patients; (5) normal coagulation function; (6) stable vital signs allowing participation in repeated behavioral, EEG, fNIRS, and taVNS assessments.

Exclusion criteria: (1) neurodegenerative diseases, postoperative coma due to intracranial infection, or brain tumors; (2) severe systemic complications; (3) participation in other clinical trials within the previous 3 months; (4) a history of severe allergy or allergic constitution; (5) unstable cardiovascular disease, clinically significant arrhythmia, severe bradycardia, uncontrolled hypertension or hypotension, implanted pacemaker or other implanted electrical device, or severe autonomic instability; (6) uncontrolled epilepsy or other conditions judged by the investigators to make taVNS unsafe. Stable cardiovascular or blood pressure conditions that do not meet exclusion criteria will be recorded at baseline and considered in sensitivity or covariate-adjusted analyses.

### 5.4 taVNS protocol

All enrolled patients will receive taVNS treatment in addition to standard rehabilitation. The taVNS intervention will last for 4 weeks and will be delivered using a transcutaneous electrical nerve stimulator (SDZ-IIB, Suzhou Medical Supplies Factory). Stimulation will be applied bilaterally to the auricular concha region, including the cymba conchae and cavum conchae. During stimulation, a pair of identical dedicated stimulation clips will be attached to both ears. Each clip is equipped with three silicone electrode terminals: one serves as the common electrode and is positioned behind the auricle, while the other two are placed at the cavum conchae and cymba conchae, respectively.

The stimulation parameters will be set to a dilatational wave with alternating frequencies of 4/20 Hz, a pulse width of 200 microseconds, and a current amplitude range of 1.0 to 1.5 mA. Unless contraindicated by vital signs or discomfort indicators, the routine stimulation intensity will be set at 1.5 mA. taVNS will be administered twice daily, once in the morning and once in the afternoon, with each session lasting 30 min. The treatment schedule will follow a cycle of 5 consecutive treatment days followed by 2 days of rest, for a total duration of 4 weeks. The stimulator will have no ramp-up or ramp-down period during stimulation.

Before each stimulation session, the condition of the auricular skin and the placement of the electrodes will be checked to ensure good electrode contact and treatment safety. Because patients with DOC cannot reliably report subjective sensation, stimulation tolerance will be determined using objective bedside indicators, including oxygen saturation, heart rate, blood pressure, facial expression, motor response, Nociception Coma Scale-Revised (NCS-R) score, arousal fluctuation, autonomic signs, and local skin reaction. The current intensity will be reduced or the session will be stopped if oxygen saturation falls below 95%, heart rate increases by more than 20% compared with the pre-stimulation level, NCS-R score exceeds 3 points with a clear increase from baseline, clinically relevant blood pressure fluctuation, bradycardia, arrhythmia, seizure-like activity, marked autonomic instability, skin irritation, or any other adverse event occurs. All taVNS procedures will be performed by trained research staff or rehabilitation therapists.

Standard rehabilitation will be provided according to the routine DOC rehabilitation program at the study center and will include standard medical and nursing care, positioning and range-of-motion training, respiratory management, nutritional support, sensory stimulation, and physical or occupational rehabilitation as clinically indicated. To reduce confounding, the type, frequency, duration, and major changes in standard rehabilitation, as well as medication changes that may affect arousal, will be prospectively recorded during the study and considered in statistical analyses.

Adverse events will be monitored throughout the intervention period. Expected adverse events include local auricular skin redness or irritation, discomfort-related behavioral responses, transient autonomic fluctuation, bradycardia, arrhythmia, blood pressure instability, oxygen desaturation, and seizure-like events. Serious adverse events will be reported to the ethics committee according to institutional requirements. Participants may discontinue stimulation temporarily or permanently if investigators judge that continued stimulation may increase clinical risk.

## 6 Procedure and data collection

### 6.1 Inclusion procedure

Patients with DOC will be recruited from the Neurosurgical Ward for DOC at Beijing Tiantan Hospital, Capital Medical University. Before study initiation, the investigators will provide the patients’ legal representatives with a detailed explanation of the study objectives, procedures, potential benefits and risks, methods of data collection and management, as well as the rights of the participants and their legal representatives, including the right to withdraw from the study at any time without affecting routine medical care. Written study information will be provided to the legal representatives. As patients with DOC are usually unable to provide informed consent independently, written informed consent must be signed by the legal representative of each participant before enrollment. After informed consent is obtained, the investigators will assess eligibility according to the predefined inclusion and exclusion criteria. Demographic and clinical data will then be collected, and baseline assessments will be completed before the initiation of taVNS treatment, including CRS-R scoring and data acquisition using EEG and fNIRS. This study is a single-center, prospective, exploratory clinical study. All enrolled patients will receive taVNS treatment in addition to standard rehabilitation, and all participants will undergo CRS-R assessment, EEG recording, and fNIRS data acquisition at baseline, week 2, and week 4 of treatment.

### 6.2 Overview of study

For an overview of the intervention, assessment tools, and assessment time points, please refer to Figures 1, 2.

**Figure 1:**
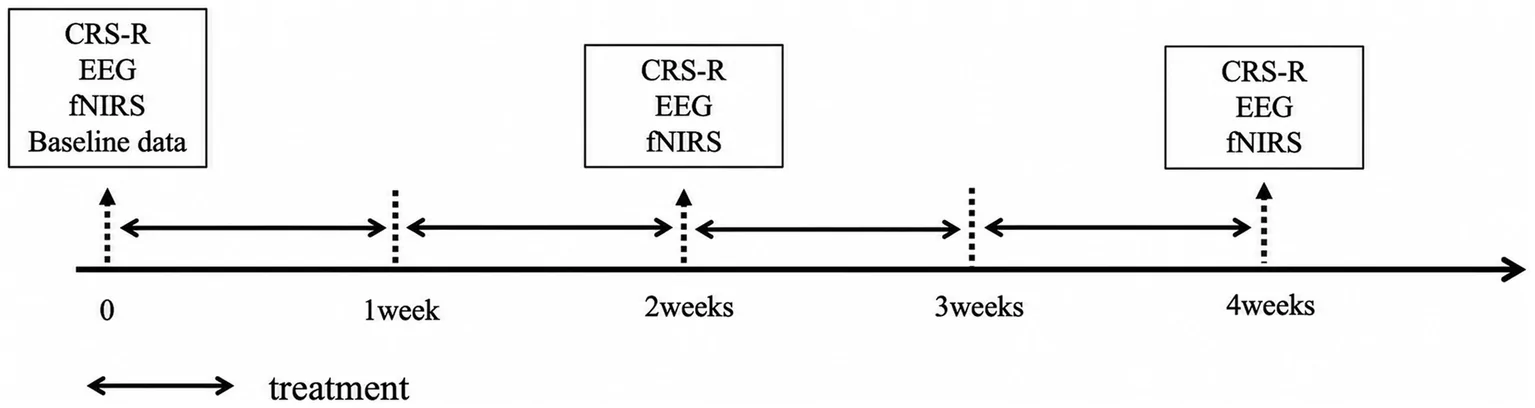
Overview of the intervention.

**Figure 2:**
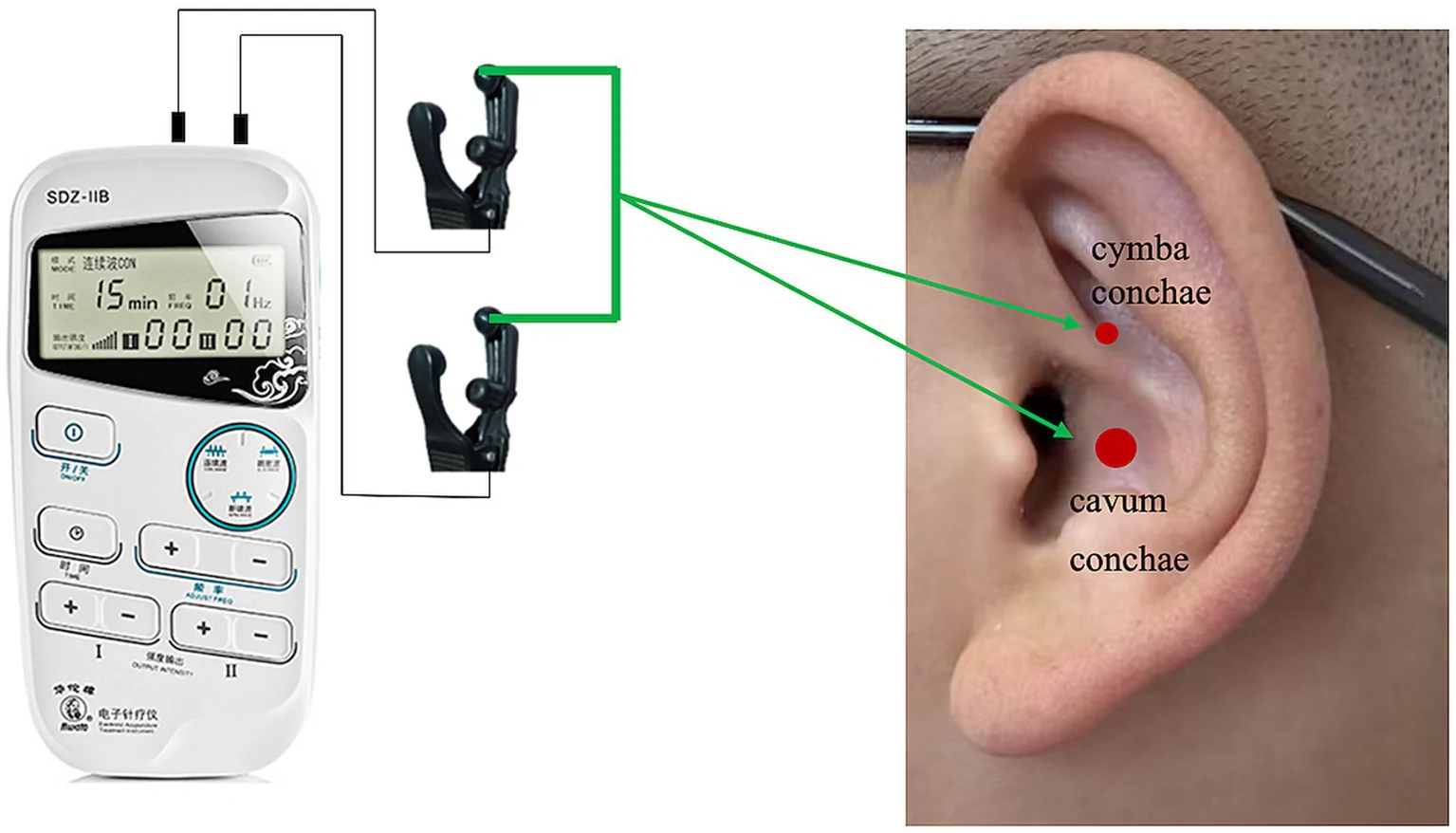
TaVNS treatment.

### 6.3 Behavioral assessment

CRS-R assessments will be performed at baseline, week 2, and week 4 by trained evaluators. To reduce the influence of arousal fluctuation, assessments will be conducted under stable clinical conditions and, when feasible, repeated observations will be used to confirm clinically meaningful changes. Responder status will be determined using an *a priori* operational threshold, defined as an increase of ≥3 points in the CRS-R total score from baseline to week 4. This threshold is not intended to represent a validated minimal clinically important difference for patients with DOC or for taVNS treatment. Rather, it was selected to provide a conservative dichotomization of observed behavioral improvement in this exploratory protocol and to reduce the likelihood that small CRS-R fluctuations or transient arousal changes would be classified as treatment response.

### 6.4 EEG recording and preprocessing

Resting-state EEG will be recorded for 30 min at each assessment time point using 32 Ag/AgCl electrodes (Nicolet EEG V32, Natus Neurology, United States) placed according to the international 10–20 system. The sampling rate will be 1,000 Hz, and electrode impedance will be maintained below 5 kΩ. EEG recordings will be performed before taVNS stimulation at each assessment time point, with patients in a quiet resting condition and without active stimulation. If the patient becomes fatigued or falls asleep during recording, EEG monitoring will be paused and the patient will be kept awake by gentle stimulation of the earlobe before recording resumes. Quantitative EEG analysis will require at least 5 min of artifact-free resting-state data after preprocessing.

EEG data will be preprocessed offline in MATLAB (Version 2020b, MathWorks Inc., Natick, MA, United States). Preprocessing will include 2–20 Hz band-pass filtering, 50 Hz notch filtering, down-sampling to 250 Hz, removal of ocular, muscular, cardiac, and other non-neural artifacts using independent component analysis, and re-referencing to the average reference. The continuous EEG data will be segmented into non-overlapping 2-s epochs. Bad channels and epochs will be identified using visual inspection and automated artifact-detection criteria. Channels with persistent noise in more than 20% of epochs will be excluded or interpolated, and EEG datasets with more than 25% bad channels or fewer than 150 artifact-free 2-s epochs will be excluded from quantitative analyses.

Spectral power will be calculated for the following predefined frequency bands: delta, 2–4 Hz; theta, 4–8 Hz; alpha, 8–13 Hz; and beta, 13–20 Hz. Functional connectivity will be quantified primarily using the weighted phase lag index, with magnitude-squared coherence calculated as a supplementary connectivity metric. Network measures derived from the connectivity matrices will include global efficiency, local efficiency, clustering coefficient, characteristic path length, and nodal degree/strength. Network complexity will be assessed using normalized Lempel-Ziv complexity and sample entropy, with sample entropy calculated using an embedding dimension of 2 and a tolerance of 0.2 times the standard deviation of the signal. EEG microstate analysis will be performed using global field power peaks and modified k-means clustering with four canonical microstate classes. Microstate parameters will include global explained variance, mean duration, occurrence, temporal coverage, and transition probabilities.

### 6.5 fNIRS acquisition and preprocessing

Hemodynamic data will be acquired using the NIRScout system (NIRx Inc.). The system uses two wavelengths, 760 and 850 nm, to distinguish changes in deoxygenated and oxygenated hemoglobin concentrations. Optodes will be arranged according to the international 10–20 system. Specifically, 16 sources and 16 detectors will be placed over the prefrontal cortex, primary motor cortex, and primary sensorimotor cortex to yield 48 optical channels. The source-detector distance will be 30 mm, and the sampling rate will be 7.81 Hz. Resting-state fNIRS data will be recorded for 10 min at each assessment time point before taVNS stimulation, with patients in a quiet resting condition and with external sensory stimulation minimized.

fNIRS data will be analyzed using HOMER2 and MATLAB (Version 2020b, MathWorks Inc., Natick, MA, United States). Raw light intensity data will first be converted to optical density. Motion artifacts will be detected using HOMER2 channel-wise motion-detection procedures and corrected using spline interpolation and wavelet-based motion correction. The corrected signals will then be band-pass filtered at 0.01–0.08 Hz to retain low-frequency resting-state hemodynamic fluctuations. Optical density data will be converted into relative changes in oxygenated hemoglobin and deoxygenated hemoglobin using the modified Beer–Lambert law.

Signal quality will be assessed for each optical channel before feature extraction. Channels will be excluded if they show saturated or extremely low raw intensity, scalp coupling index <0.80, coefficient of variation>15%, source-detector distance outside the predefined acceptable range, or motion artifacts affecting more than 20% of the recording after correction. Participants with fewer than 5 min of usable artifact-free fNIRS data or with fewer than 70% valid channels in the predefined regions of interest will be excluded from quantitative fNIRS analyses. Planned fNIRS features will include regional HbO and HbR response amplitude, resting-state hemodynamic fluctuation amplitude, and functional connectivity among the recorded cortical regions. Functional connectivity will be calculated using Pearson correlation coefficients between HbO time series and then transformed using Fisher’s z transformation; HbR-based connectivity will be analyzed as a supportive measure.

## 7 Outcomes and instruments

### 7.1 Primary outcome measure

The observed change in the CRS-R total score from baseline to the end of the 4-week taVNS intervention.

### 7.2 Secondary outcome measures

1) Changes in CRS-R total scores and subscale scores at baseline, week 2, and week 4;
2) Post-treatment diagnostic transitions in the state of consciousness, including transitions from UWS/VS to MCS-, or from UWS/VS or MCS- to MCS+;
3) Changes in prespecified EEG features between the responder and non-responder groups, including spectral power, functional connectivity, network complexity, and microstate parameters;
4) Changes in prespecified fNIRS features between the responder and non-responder groups, including HbO and HbR responses, cortical activation patterns, and functional connectivity.

## 8 Statistical analyses

This study is a single-center, prospective, exploratory clinical study designed to characterize behavioral changes during a 4-week course of taVNS plus standard rehabilitation and to screen prespecified EEG and fNIRS features associated with observed clinical improvement. Because reliable prior estimates are unavailable for the combined behavioral, EEG, and fNIRS outcomes, no formal efficacy-based sample size calculation was performed. A total of 50 patients will be enrolled based on the exploratory nature of the study and recruitment feasibility at the study center. Assuming an approximately 15% rate of loss to follow-up or incomplete behavioral, EEG, or fNIRS data, approximately 43 participants are expected to contribute analyzable data. The biomarker analyses are intended for hypothesis-driven feature screening and hypothesis generation, and no multivariable clinical prediction model will be developed or validated.

Statistical analyses will be performed using SPSS 26.0 and MATLAB, as appropriate. All tests will be two-sided, and *p* < 0.05 will be considered statistically significant. Categorical variables will be presented as frequencies and percentages. Continuous variables will be expressed as mean ± standard deviation if normally distributed or as median and interquartile range if not normally distributed.

### 8.1 Primary outcome analysis

The primary outcome is the observed change in the CRS-R total score from baseline to week 4. Normality will first be assessed using the Shapiro–Wilk test. If the data are normally distributed, a paired t-test will be used; otherwise, the Wilcoxon signed-rank test will be applied. In addition, a linear mixed-effects model with time as a fixed effect and participant as a random effect will be used to evaluate longitudinal CRS-R changes across baseline, week 2, and week 4, with adjustment for clinically relevant covariates when appropriate.

### 8.2 Secondary outcome analysis

Changes in CRS-R total scores and subscale scores across baseline, week 2, and week 4 will be analyzed using repeated-measures analysis of variance for normally distributed data, or the Friedman test for non-normally distributed data, followed by *post hoc* pairwise comparisons. Longitudinal analyses will also be performed using mixed-effects models to account for repeated measurements and incomplete follow-up data. Diagnostic transitions in consciousness state will be described using frequencies and percentages. Patients will be classified into a responder group and a non-responder group according to whether the CRS-R total score at week 4 shows an improvement of ≥3 points compared with baseline. Sensitivity analyses will additionally examine diagnostic transition and any CRS-R improvement greater than 0 as alternative response definitions. Baseline characteristics between the two groups will be compared using the independent-samples t-test or Mann–Whitney U test for continuous variables, and the chi-square test or Fisher’s exact test for categorical variables. Age, sex, etiology, disease duration, baseline diagnosis, baseline CRS-R score, stable cardiovascular or blood pressure conditions, medication changes, and rehabilitation exposure will be considered as covariates where appropriate. Changes in EEG and fNIRS measures across the three time points will be analyzed using repeated-measures models or mixed-effects models, and comparisons between the responder and non-responder groups will be performed using parametric or non-parametric tests as appropriate. Correlations between changes in CRS-R scores and changes in EEG/fNIRS measures will be assessed using Pearson or Spearman correlation analyses. False discovery rate correction will be applied for multiple comparisons across EEG and fNIRS features. Missing-data patterns and the reasons for missingness will be documented and summarized by assessment time point and data modality. The primary mixed-effects analyses will assume that data are missing at random. For dropout plausibly related to clinical deterioration and therefore potentially missing not at random, participants with missing week-4 responder status will be conservatively classified as non-responders, and delta-adjusted pattern-mixture sensitivity analyses will be performed for continuous CRS-R outcomes under progressively unfavorable assumptions. The results of these sensitivity analyses will be compared with those of the primary analyses to assess the robustness of the conclusions.

To distinguish hypothesis-driven analyses from exploratory analyses, EEG and fNIRS features will be organized into prespecified primary candidate biomarkers and exploratory features before statistical analysis. The primary candidate biomarker set will be based on the study hypotheses and will include: (1) EEG relative theta and alpha power over frontal and parietal regions; (2) EEG frontoparietal functional connectivity measured using the weighted phase lag index in the theta and alpha bands; (3) EEG global efficiency and normalized Lempel-Ziv complexity; and (4) fNIRS regional HbO amplitude and HbO functional connectivity among the prefrontal, motor, and sensorimotor regions. These features will be analyzed first as the main candidate biomarkers associated with observed CRS-R improvement.

Other EEG and fNIRS measures, including delta and beta band features, supplementary connectivity metrics, additional graph-theoretical measures, sample entropy, microstate parameters, HbR-based measures, and channel-wise or region-wise features not included in the primary candidate biomarker set, will be treated as exploratory features. Results from exploratory analyses will be interpreted as hypothesis-generating.

## 9 Discussion

This study is designed as a single-center, prospective, exploratory clinical study aimed at characterizing behavioral changes during taVNS plus standard rehabilitation in patients with DOC and exploring multimodal brain functional biomarkers associated with observed response by integrating EEG and fNIRS. As a non-invasive neuromodulation technique, taVNS has attracted increasing attention in the field of DOC in recent years because of its relatively high safety, ease of operation, repeatability, and comparatively low treatment cost (Shou et al., 2021). Compared with invasive neuromodulation approaches such as SCS and DBS, taVNS has greater clinical accessibility and may provide a more easily scalable arousal-promoting intervention for patients with prolonged DOC (Wan et al., 2024).

The rationale for this study is that taVNS may modulate neural networks related to arousal and consciousness by stimulating the auricular vagal afferent pathway and influencing the brainstem-thalamic-cortical system (Wang L. et al., 2023). Previous studies have suggested that taVNS can improve behavioral scores and facilitate diagnostic transitions in a subset of patients with DOC (Wang Y. et al., 2024b). However, there is marked interindividual variability in treatment response, and not all patients appear to benefit from this intervention. Therefore, in addition to using CRS-R to evaluate clinical behavioral changes, this study will further integrate EEG and fNIRS to identify objective brain functional features associated with observed response from the perspectives of electrophysiological activity and cerebral hemodynamics, with the aim of more comprehensively characterizing residual brain function and treatment-related changes.

An important strength of this study is the combination of behavioral assessment with multimodal neurofunctional evaluation and repeated assessment throughout the treatment course (Coleman et al., 2009). In patients with DOC, reliance on bedside behavioral examination alone may underestimate the level of residual consciousness, particularly in those with severe motor impairment, fluctuating arousal, or restricted sensorimotor output (Monti et al., 2009). EEG offers high temporal resolution and strong bedside applicability, and can be used to assess brain functional features such as spectral power, functional connectivity, network complexity, and microstate dynamics (Galiotta et al., 2022). fNIRS can complement these measures by providing information on cortical hemodynamics, including HbO/HbR responses, regional activation patterns, and functional connectivity (Abdalmalak et al., 2021). By combining CRS-R, EEG, and fNIRS, this study is expected to establish a more comprehensive exploratory evaluation framework for identifying candidate markers of observed response during taVNS plus standard rehabilitation.

This study has several limitations. First, as a single-center exploratory study with a planned sample of 50 patients, the statistical precision and generalizability of the findings will be limited, and the biomarker analyses are intended primarily for feature screening and hypothesis generation. Most importantly, the absence of a sham-stimulation or standard-care-only control group precludes causal attribution of changes in CRS-R, EEG, or fNIRS measures to taVNS. Patients with DOC may experience spontaneous recovery and natural fluctuations in arousal, while concurrent rehabilitation, medication adjustments, intercurrent clinical events, and repeated assessments may also influence the observed outcomes. Therefore, the candidate biomarkers identified in this study are features associated with clinical improvement during taVNS plus standard rehabilitation, rather than biomarkers of taVNS-specific efficacy. Medication exposure and rehabilitation intensity will be prospectively documented and included in adjusted and sensitivity analyses; however, residual and unmeasured confounding cannot be eliminated. In addition, heterogeneity in etiology, disease duration, baseline consciousness level, and comorbidities may further increase interindividual variability. Confirmation of biomarkers specifically associated with taVNS efficacy will require future randomized sham-controlled or standard-care-controlled studies.

Despite these limitations, this study is still expected to provide important preliminary and hypothesis-generating evidence for the clinical evaluation of taVNS in DOC and for understanding brain functional features associated with observed response. If EEG and fNIRS features associated with behavioral improvement can be identified, these measures may serve as candidate objective biomarkers for screening patients who are more likely to show improvement during taVNS plus standard rehabilitation. In addition, the results of this study may provide a basis for future large-sample randomized controlled trials and lay the foundation for establishing individualized and objective neuromodulation strategies for patients with DOC (Wang et al., 2024a).
